# Olive Oil in Large Doses in Gall-Stone Disease

**Published:** 1867-08

**Authors:** Ira Hatch

**Affiliations:** Chicago, Ill.


ARTICLE XXXIII.

### OLIVE OIL IN LARGE DOSES IN GALL-STONE DISEASE.

BY IRA HATCH, M.D., Chicago, Ill.

Read to the Chicago Medical Society.

On the 18th of last May, I was called to see Mrs. K., of Evanston, whose health had been failing for a long time. She was then under the treatment of Dr. SMALL, of Chicago, who had treated her for scirrhus of the liver. She had suffered paroxysms of the most excruciating pain, with nausea, vomiting, and sometimes hiccough. These paroxysms increased in severity and frequency. The treatment failed to relieve even the pain, much less remove the cause, and she was forced, ultimately, to resort to anodynes. She took 60 drops of McMunn's elixir at a dose, which relieved the pain while the patient was under its influence. In the meantime, she was growing weaker, and the case was becoming desperate.

The following were the most prominent symptoms when I first saw her:—Skin as yellow as an orange; bowels obstinately constipated; countenance sunken and haggard; violent pain in the epigastric region, running around the right side nearly to the spine; nausea and vomiting; extreme thirst, and distress for breath, apparently from defective innervation. It was evident that the jaundice, pain, constipation, and vomiting were owing to the presence of gall-stones.

She was advised to take large doses of olive oil, to soften and expel the gall-stones. She resolutely determined to make the effort, though it seemed extremely doubtful about keeping the oil on the stomach; but she succeeded, by the help of a little cider taken after the oil. The first night she took a half bottle, and the two following nights the remainder of the bottle of oil. It operated freely and brought off a great number of gall-stones, resembling full-grown gooseberries, in size and color. As they floated upon the top of the water, they were mistaken and thrown out; but enough were saved to prove their character. They were soft and completely saturated with the oil. she was greatly relieved by the operation. But, three or four days afterwards, fancying that the old pain was about to return, she repeated the oil, and came near sinking under the operation. It increased the exhaustion and irritability of the mucous membranes of the stomach and duodenum. Appearances were decidedly against her. Although the cause of the disease was removed, certain consequences which might prove fatal. The most prominent of these were, irritation, if not inflammation of the mucous membranes of the stomach and duodenum

To remove this irritation, or sub-acute inflammation of the mucous surface, seemed to be the principal indication for treatment. All solid food was forbidden. She had craved and had taken some solid food, which had probably kept up the pain after the gall-stones were evacuated. To allay the thirst, which was intense, she took small quantities of Vichy water, ice-cold, every few minutes. This was grateful to the stomach, it relieved the nausea and sicknese. She also took the white silver mixture, prepared as follows:—

℞. Argent. Nitratis,-------------------- grs. xvi.
Potassii Iodidi,-------------------- grs. xxiv.
Aqua Distil.,---------------------- ℥iv.

Dissolve the nitrate of silver in the water, add of the iodide sufficient to form a pale yellow precipitate; let it settle, pour off the water and supply its place with pure water, then add the remainder of the iodide of potassa. Shake well, and take a teaspoonful every four hours.

After two days she could bear a weak, cold infusion of the hydrastis Canadensis, which acts well in certain forms of subacute inflammation of the mucous membrane.* The infusion of the root should be weak, or it will offend; especially where there is excessive irritation.

Mrs. W., a sister and neighbor of Mrs. K., was laboring under the same disease, but in a less aggravated form. Her skin was very yellow, bowels constipated, and appetite poor;

* I have never seen any preparation of the hydrastis that has satisfied me as well as the infusion of the root in cold water.

she had every appearance of gall-stones. What heightened the probability was, that her mother and oldest sister died, a few years since, of the same diaease. Encouraged by the success in Mrs. K.'s case, she took the oil; one bottle in four days. It did not operate till the whole was taken, and then the operation was mild, and the gall-stones, in large quantities, passed without pain. She is now recovering her health surely and rapidly.

I saved a few of the calculi to exhibit to the Society; they were then in good shape, but soft, and perfectly saturated with the oil. I placed two of them upon a paper and exposed them to the heat of the sun, to harden, but they melted down, completely dissolved in the oil; thus demonstrating the fact that biliary calculi are completely soluble in olive oil.

I do not ktow of any author who has ever hinted at the possibility of dissolving these calculi in the living body. DUNGLISON says, "solvents are not to be depended upon. They cannot reach the calculi." Dr. KIDD, of London, praises chloroform highly, in gall-stone colic. He argues that it lets through the calculi, by relaxing the ducts; but he seems to have no notion of a solvent. Dr. THUDICUM believes that their solution in the living body is impossible. But the history of the two cases above related, shows conclusively that biliary calculi are soluble in olive oil, while in the gall-bladder of the living subject.

These concretions seem to be composed mostly of inspissated bile, hardened around a small deposit of cholesterine, which seemed to serve as a nucleus. In the centre of each calculus that I examined, there was a whitish substance, resembling tallow softened by oil. The oil had completely permeated every particle of the concretions.

